# Tracing the origins of glioblastoma by investigating the role of gliogenic and related neurogenic genes/signaling pathways in GBM development: a systematic review

**DOI:** 10.1186/s12957-022-02602-5

**Published:** 2022-05-10

**Authors:** Ovais Shafi, Ghazia Siddiqui

**Affiliations:** Sindh Medical College - Jinnah Sindh Medical University / Dow University of Health Sciences, Karachi, Pakistan

**Keywords:** Glioblastoma, Gliogenesis, Aging, Neurogenesis, Glioblastoma origins, Inflammation, Cancer, Oncogenesis, Stem cells, Regeneration

## Abstract

**Background:**

Glioblastoma is one of the most aggressive tumors. The etiology and the factors determining its onset are not yet entirely known. This study investigates the origins of GBM, and for this purpose, it focuses primarily on developmental gliogenic processes. It also focuses on the impact of the related neurogenic developmental processes in glioblastoma oncogenesis. It also addresses why glial cells are at more risk of tumor development compared to neurons.

**Methods:**

Databases including PubMed, MEDLINE, and Google Scholar were searched for published articles without any date restrictions, involving glioblastoma, gliogenesis, neurogenesis, stemness, neural stem cells, gliogenic signaling and pathways, neurogenic signaling and pathways, and astrocytogenic genes.

**Results:**

The origin of GBM is dependent on dysregulation in multiple genes and pathways that accumulatively converge the cells towards oncogenesis. There are multiple layers of steps in glioblastoma oncogenesis including the failure of cell fate-specific genes to keep the cells differentiated in their specific cell types such as p300, BMP, HOPX, and NRSF/REST. There are genes and signaling pathways that are involved in differentiation and also contribute to GBM such as FGFR3, JAK-STAT, and hey1. The genes that contribute to differentiation processes but also contribute to stemness in GBM include notch, Sox9, Sox4, c-myc gene overrides p300, and then GFAP, leading to upregulation of nestin, SHH, NF-κB, and others. GBM mutations pathologically impact the cell circuitry such as the interaction between Sox2 and JAK-STAT pathway, resulting in GBM development and progression.

**Conclusion:**

Glioblastoma originates when the gene expression of key gliogenic genes and signaling pathways become dysregulated. This study identifies key gliogenic genes having the ability to control oncogenesis in glioblastoma cells, including p300, BMP, PAX6, HOPX, NRSF/REST, LIF, and TGF beta. It also identifies key neurogenic genes having the ability to control oncogenesis including PAX6, neurogenins including Ngn1, NeuroD1, NeuroD4, Numb, NKX6-1 Ebf, Myt1, and ASCL1. This study also postulates how aging contributes to the onset of glioblastoma by dysregulating the gene expression of NF-κB, REST/NRSF, ERK, AKT, EGFR, and others.

## Background

The etiology and origins of glioblastoma (GBM) are still unknown. GBM is unique in many ways as 90% of the time it occurs as a primary tumor and 10% of the time as a secondary tumor. It is more common in the elderly population. The development of GBM is considered to be very complex in nature. Despite the fact that multiple genes and signaling pathways have been identified, still the origins of GBM remain largely unknown [1].

Neural stem cells (NSCs) are progenitors of both neurons and glial cells, but the risk of tumor development is entirely different in both cell types. Glial cells are considered to be more involved in one of the most aggressive CNS tumor, i.e., GBM, but there are comparatively very few tumors of neuronal origin. It is important to note that medulloblastoma which is considered to be a tumor of neuronal origin, it is mostly found in children but some cases are also found in adults till age 40. Neurons remain largely free of tumors of neuronal origin, but glial cells including astrocytes are at risk of tumor development such as GBM [2–4].

The task of this study is to trace the origins of GBM. This study investigates GBM using the developmental biology of glial cells, also by focusing on related neurogenic genes/signaling pathways in glioblastoma oncogenesis. In the light of the findings of this study, it goes on further to postulate in the ‘Discussion’ section the likely reasons why glial cells are more predisposed to oncogenesis compared to neuronal cells. This study also investigates the related aspects of gliogenesis and neurogenesis to further investigate the origins of GBM.

## Methods

PUBMED database, MEDLINE database, Google Scholar, and online journals such as BMC, PLOS, Cancer Cell, and Neoplasia were searched with no date restrictions for published articles in relation to ‘glioblastoma’, ‘gliogenesis’, ‘neurogenesis’, and ‘neural stem cells’. It also sheds light on the genetic heterogeneity in GBM development.

Keywords used to investigate the origins of glioblastoma: gliogenesis, gliogenic genes and signaling pathways, neurogenesis, neurogenic genes, and signaling pathways.

### The following gliogenic genes and signaling pathways were investigated for their role in glioblastoma oncogenesis

IL-6 family, FGFR3, JAK-STAT signaling, BMPs, Notch signaling, Notch effector protein NFIA, SOX9, SOX4, STAT3, GFAP and S100, BMP with SMAD*,* p300/CBP, Notch/hey1, HES genes interactions between STAT3 and JAK 2, SHH, PAX6 with Nkx6.1, NF-κB signaling and inflammation in gliogenesis and GBM, Neuregulin-1, neuronal restrictive silencing factor (NRSF)/REST, MAPK, MEK, TGF beta, E2F, TCFL2, p130 with JAK STAT, transcription factors NFIC and HOPX, Ephrins (EFNB1), and Netrins (NTN).

### The following related neurogenic genes and signaling pathways were investigated for their role in glioblastoma oncogenesis

Ngn1, NeuroD transcription factor, PDGF and Neurotrophin-3, CNS neural progenitor markers (*Pax7, Dbx2*, *Nkx6.1, FGF*), neurogenin-mediated neurogenesis (*Ebf2,3, Hes6, Myt1, Neurod1, Neurod4, and Runx1t1), PAX6, BMP in neuronal development, Numb gene,* ASCL1, klf4, c-myc, SOX4, SOX2 induces ASCL1 and TLX TF, wnt signaling, Notch/STAT3-Ser/Hes3 Axis, GSK3 beta.

Only those articles were eligible to be included in this study on glioblastoma which were related to gliogenic genes and signaling pathways, key genes involved in neurogenesis specifically focusing on neuronal differentiation and also contribute to glioblastoma oncogenesis. Screening of the literature was also done on the same basis and related data was extracted. The literature search began in November 2018 and ended in February 2021. During revision, further literature was searched and referenced until November 2021.

The literature search and all sections of the manuscript were checked multiple times during the months of revision (March 2021–November 2021) to maintain the highest accuracy possible. Further revisions were made in April 2022. The prime focus of the literature search was to screen the literature on the basis of the eligibility criteria mentioned above. This study is a meta-analysis. Publications only in ‘English’ were used, and there was no limitation on the date of publication. Data extraction was based on these eligibility criteria. No unpublished study was used or included. This study adheres to relevant PRISMA guidelines (Preferred Reporting Items for Systematic Reviews and Meta-Analyses).

## Results

A total of 3810 articles were identified using database searching, and 3494 were recorded after duplicates removal. Three thousand sixty-six (3066) were excluded after screening of title/abstract, 215 were finally excluded (because when many separate articles were present with similar conclusions, only those were selected to be included which mainly focused on genes/signaling pathways involved in gliogenesis and neurogenesis in relation to GBM development), and 3 articles were excluded during data extraction. Finally, 210 articles were included (based on the objectives of the study).

There are also limitations of this study as mentioned in the ‘Methodology’, ‘Study design’, and other respective sections. The TP53, RB1, MDM2, NF1, or other such factors that already have a well-established role in glioblastoma development [5], they are not the primary focus of this study.

Based on the results of this study, it also addresses in the ‘Discussion’ section the increased predisposition of glial cells towards oncogenesis compared to neurons. This will help to understand the factors that put the glial cells such as astrocytes more towards at risk of oncogenesis such as glioblastoma development.

**Fig. 1:**
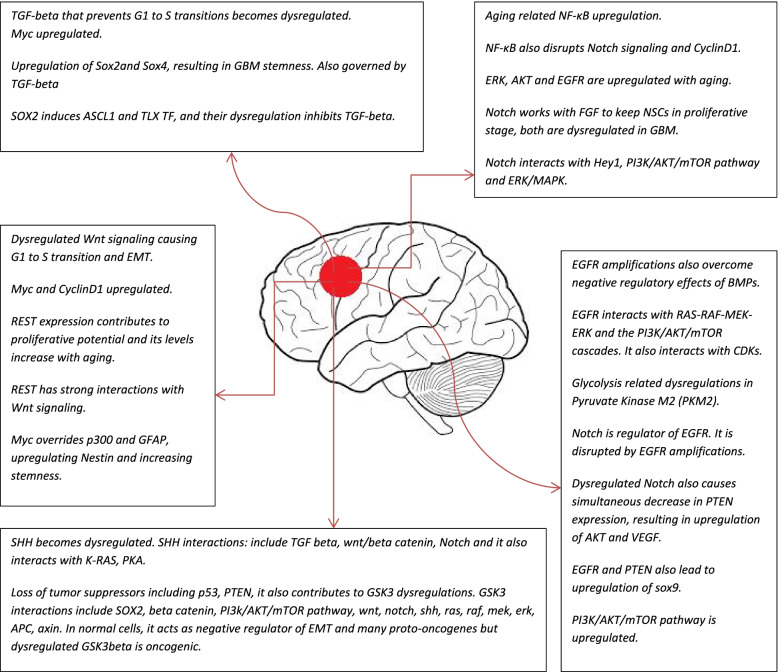
Interconnectedness of GBM landscape: This study postulates a possible sequence of key changes that unfolds and they ultimately lead to the GBM development

**Fig. 2:**
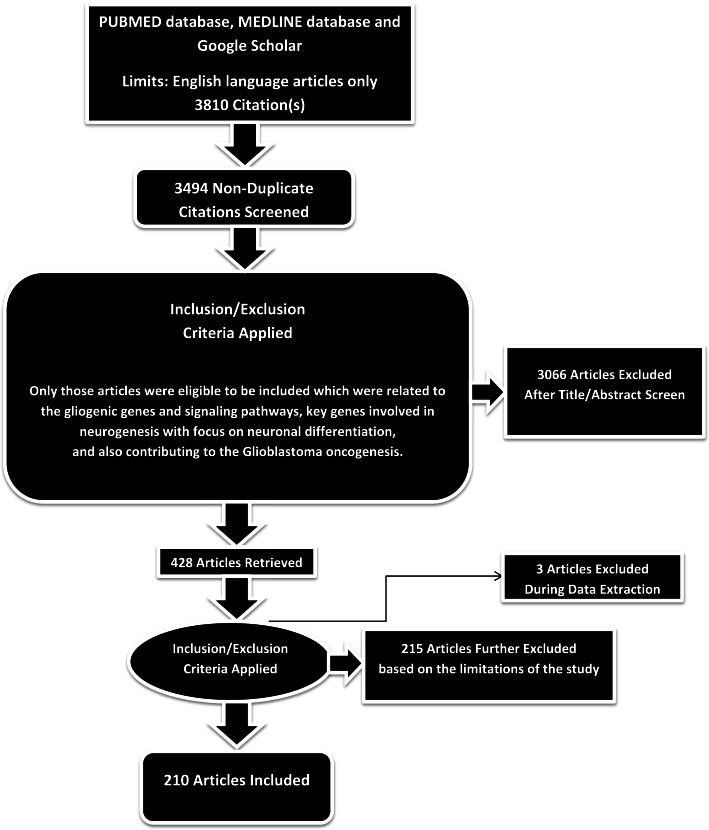
PRISMA flow diagram: This figure only highlights the methodology of the study in relation to its limitations. The limitations are detailed in the ‘Methodology’, ‘Study design’, and in the beginning of ‘Results’ sections. This figure represents graphically the flow of citations in the study

This study investigates the gliogenic genes and signaling pathways to trace the origins of GBM, and it also focuses on related neurogenic genes/signaling pathways that play role in GBM oncogenesis. The summary of the key findings is present in Tables 1 and 2. This study focuses on the signaling pathways and genes that work in the form of combinatorial codes in cell type-specific programming in gliogenesis and neurogenesis. This study also tries to map the landscape of genetic switches that lead to the origin of glioblastoma, also illustrated in Figs. 1 and 2 (landscape of GBM onset and development).

**Table 1:** Gliogenic genes/signaling pathways and their role in glioblastoma development

Genes/signaling pathways	Role in gliogenesis	Role in GBM	Comments
TGF beta including BMP	Gliogenic. BMP works with SMAD in gliogensis	Inhibit G1 to S transitions and cause cytostasis.	Inhibit c-myc. BMP downregulates EGFR.
PAX6	Neurogenic but with Nkx6.1 it contributes to astrocytogenesis	Upregulated PAX6 acts as tumor suppressor	Controls VEGF, angiogenesis, and GBM invasiveness.
P300	Gliogenic, induces the cells more towards GFAP expression.	Anti-GBM. The c-myc gene overrides p300 and then GFAP, leading to upregulation of nestin	Repressor of nestin which is also involved with stemness (sox2).
NRSF/REST	Astrocytogenic. It halts neurogenesis and induces gliogenesis.	Strong proliferative response makes it capable of contributing to GBM oncogenesis.	REST amplification is also implicated in GBM. Promotes stemness in GBM.
LIF	Gliogenic. Low levels cause differentiation.	In GBM, TGF-beta signaling causes it to become pro-oncogenic.	When LIF is applied alone to cell lines, it causes growth inhibition in GBM.
HOPX	Primarily astrocytogenic	Tumor suppressor. Downregulated in GBM	It keeps NSCs in quiescent stage.
Notch	Contributes to gliogenesis but also contribute to the stemness in GBM	EGFR and Notch are interlinked and are upregulated in GBM.	NOTCH works with FGF to keep NSCs in proliferative stage. NPCs are regulated by notch signaling.
SOX9 and SOX4	Involved in gliogenesis but in GBM micro-environment lead to stemness.	Sox members are involved in reprogramming of GBM stem cells	Interacts with pathways Shh and Notch in morphogenesis.
SHH	More neurogenic than oncogenic.	In GBM, shh upregulates Hes1 and is involved in stemness.	When PTEN becomes defective in GBM, then SHH and PI3K become dysregulated.
FGFR	Gliogenic roles but in GBM development contribute to the oncogenesis.	FGFR has strong interactions with MAPK and in GBM become dysregulated	FGFR1 is expressed in neurons and FGFR2 is more expressed in gliogenic differentiation
JAK-STAT	Gliogenic. It also contributes to the stem cell maintenance.	In GBM development, it becomes oncogenic.	Vast net of interactions. It interacts with PI3K/AKT/mTOR pathway, MAPK/ERK pathway and several others.
STAT3	Gliogenic. But micro-environment also has vast effect on its role.	In GBM, STAT3 mutations make massive contributions to GBM oncogenesis.	It also interacts with EGFR which plays key role in GBM development
IL-6 family	Gliogenic.	Works with STAT3 in promoting pro-oncogenic pathways	With age, the gene expression of inflammatory cytokines increase in the body.
G FAP and S100	Involved in astrocytogenesis	Upregulated in GBM	GFAP also regulate astrocyte neuronal interactions
Hey1	Works with Notch in gliogenesis	Dysregulated Hes1 plays role in stemness and EMT induction in GBM development.	Shh also upregulates Hes1 gene expression
NF-κB	When dysregulated, harms neural stem cells (NSCs) and gliogenesis potential.	In GBM oncogenesis, it contributes to EGFR amplification.	NF-κB in GBM also contributes to EMT and GBM stemness.
Neuregulin-1	It is a gene of EGF family and contributes to astrocytogenesis.	Nrg1 and erb interact with each other and in GBM contribute to oncogenesis.	Nrg1, TGF alpha, EGFR all have profound interactions with one another and impact PI3k/AKT pathway, MAPK and JAK/STAT pathway
MAPK	Involved in gliogenesis.	In GBM, it interacts with EGFR, mTOR/PI3K/Akt, PDGFR, and RAS.	MAPK is also involved In Insulin resistance.
MEK and E2F	Involved in gliogenesis but they contribute to G1 to S transitions in GBM development.	RAS over-expresses MEK in GBM. Rb1 interacts with CDKs in inhibiting E2F.	This pathway becomes damaged in GBM and CDKs cause E2F based G1 to S transitions.
TCFL2/LEF	Involved in gliogenesis.	They have been detected in GBM samples.	These transcription factors of WNT signaling works with beta catenin.
Ephrins and Netrins	Involved in gliogenesis. Their role also includes angiogenic activity.	Their dysregulations are involved in GBM development.	NTNs contribute to GBM stemness. NTN-1 activates Notch and interacts with EGFR.
Transcription factors NFIX	NFIX TF interacts with STAT3 and is involved in gliogenesis.	NFIX works with Ezrin protein, both are dysregulated in GBM	TF NFIX regulates NPC differentiation.

**Table 2:** Related neurogenic genes/signaling pathways and their role in GBM oncogenesis

Genes/signaling pathways	Role in neurogenesis	Role in GBM	Comments
Ngn1	Involved in neurogenesis	In GBM, its expression causes mitotic arrest	It prevents gliogenesis and promotes neuronal differentiation.
NeuroD	Involved in neuronal differentiation.	In GBM, its induced gene expression also blocks the proliferation.	Their upregulation causes arrest of cell cycle in GBM cells.
BMP	Involved in both gliogenesis and neurogenesis	BMP upregulation is considered to play role in halting GBM progression	Play a major role in switching NSCs towards astrocytogenesis
PAX6	Neurogenic but with Nkx6.1 it contributes to astrocytogenesis	Upregulated PAX6 acts as tumor suppressor	Controls VEGF, angiogenesis and GBM invasiveness.
Numb	Numb gene negates Notch signaling and contributes to neuronal differentiation.	Upregulation halts GBM growth and progression. Highly upregulated in mesenchymal GBM cells	But still its tumor suppressor role is controversial
Nkx6.1	Neurogenic.	It suppresses tumor development and metastasis	PAX6 is neurogenic in development but with Nkx6.1 it contributes to astrocytogenesis
ASCL1	Strongly neurogenic	It also suppresses oncogenesis in GBM cells	In GBM, it switches the cells towards neuronal cell fate
Ebf	Neurogenic.	Its loss in GBM contributes to oncogenesis	EBF3 downregulates the gene expression of proliferation and survival related genes.
Myt1	Neurogenic transcription factor	In GBM, its expression is involved in downregulating proliferation.	Low Myt1 expression is involved in glioblastoma.
PDGF and NT3	Both are neurogenic	In GBM landscape, they play oncogenic role.	PDGFR expression is increased in all grades of glioma. NT3 upregulated.
Pax7	CNS neural progenitor marker	Pax7 becomes upregulated in GBM with PTEN deficiency.	Pax7 is involved in GBM invasiveness and oncogenic transformation of NSCs.
Dbx2	CNS neural progenitor marker	High DBX2 in GBM is linked with low survival.	Dbx2 works with REST in GBM proliferation.
Hes6	Neurogenic	Upregulated in GBM. Interacts with p53, NF-κB and c-myc.	Involved in angiogenesis, proliferation and migration.
Runx1 and Runx2	Neurogenic	In GBM, there role is oncogenic.	Oncosuppressive role is controversial.
Wnt	It is kept in check by tumor suppressors and is more involved in neurogenesis.	Dysregulated wnt signaling causes activation of CyclinD1 and c-myc, causing G1 to S phase transition	It also contributes to epithelial to mesenchymal transitions.
GSK3beta	In normal cells, it acts as negative regulator of epithelial-mesenchymal-transitions (EMTs) and many proto-oncogenes	Dysregulated GSK3beta is oncogenic.	In GBM, dysregulated GSK3-beta also acts to downregulate BMP that has significant gliogenic roles
Myc	It is also involved in neurogenesis but is oncogenic in GBM development	The myc gene overrides p300 and then GFAP, leading to upregulation of nestin. This plays very important role in GBM oncogenesis.	GSK3beta also works with PI3K/FGF signaling pathway and contributes to stability of c-Myc.
SOX2 and Sox4	Regulation and maintenance of neural stem cells	In GBM, stemness is mediated by SOX2 and SOX4	Nanog, oct4, myc, they are also major contributors to GBM stemness
Klf4	Contributes to neurogenesis and pluripotency.	Acts like an oncogene in GBM	Klf4 is involved in GBM heterogeneity and GBM stem cells development.
TLX TF	One of the key regulators of NSCs maintenance	In GBM, it leads to the progression of oncogenesis	It inhibits TGF-beta as TGF beta causes cytostasis.
Oct4	Involved in pluripotency and stemness	Its expression is several folds upregulated in GBM	Without FGF signaling, the NPCs can revert back to ESCs like state with predominant oct-4 expression.
Notch/STAT3-Ser/Hes3 Axis	Neurogenic axis. Regulator of NSCs.	In GBM, it impacts the cascades of downstream signaling pathways.	It is also linked to cancer development and diabetes type 2.

### Gliogenic genes/signaling pathways and their role in glioblastoma development

*Key gliogenic genes having the ability to control oncogenesis in glioblastoma cells:* p300, BMP, PAX6, HOPX, NRSF/REST, LIF, and TGF beta.

- **TGF beta:** It has a very important gliogenic effect. TGF beta family also includes BMP. The SMADS is working with TGF beta, and they inhibit G1 to S transitions and cause cytostasis. They also inhibit Myc [6–11].
  **BMP:** BMP-mediated PS-SMAD1/5/8 plays a significant gliogenic role. BMPs switch the progenitor cells towards gliogenesis. It also downregulates the gene expression of EGFR. The CTNF-, BMP-, and JAK-STAT-mediated astroglial differentiation is disrupted in the GBM development. The role of BMP signaling is anti-oncogenic as it causes the cell cycle to exit. CTNF and BMP dysregulations have downstream effects [12, 13]. It inhibits proliferation in GBM, but stemness remains intact [14]. Several studies have found that BMP signaling has a cell differentiation effect in GBM [15]. BMP also works with STAT3 for astroglial differentiation. This regulation of STAT3 is very crucial as STAT3 over-activation is involved in GBM oncogenesis [12].
  **BMP with SMAD proteins:** They contribute to gliogenesis in the CNS development. The SMAD/TGF-beta together regulates the cell cycle and cause cytostasis by downregulating gene expression of c-Myc. This prevents G1 to S transitions [6]. The importance of dysregulations in the regulators of the cell cycle can also be best estimated by the fact that SMAD/TGF beta becomes oncogenic in GBM and fails to prevent G1 to S phase transition. The dysregulations in SMAD alter the role of PDGF-B in GBM development. When Smad, PI3K, and FoxG1 signaling pathways are dysregulated, they switch the TGF-beta to become oncogenic [16]. EGFR-Akt-Smad signaling is also another way via which SMAD contributes to GBM development. TGF-β1 works with Smad, p38 MAPK, and PI3K/Akt signaling pathways in GBM development [17]. TGF beta interacts with VEGF and FGF. This process is negatively regulated by the PI3K pathway which is an inhibitor of FoxO localization in the nucleus. PI3K also becomes dysregulated in GBM development [18].
- **PAX6 is neurogenic in development but with Nkx6.1 it contributes to astrocytogenesis:** PAX6 role is fascinating at many levels as it controls the cell proliferation and also plays role in regulating the cell cycle. There is an inverse relationship of PAX6 with GBM development. When PAX6 is upregulated in GBM, it acts as a tumor suppressor [19, 20]. It is the combinatorial effect of multiple genes working at specific timings that regulate the establishment and homeostasis of the cell fate. PAX6 is primarily neurogenic and works with Nkx6.1 in establishing some types of astrocytes [21]. PAX6 also suppresses the invasiveness of GBM and controls the expression of matrix metalloproteinase-2 gene [22]. PAX6 also controls the VEGF expression in GBM and prevents angiogenesis. It stops the cells from entering the S-phase of the cell cycle and also induces apoptosis. It also interacts with PTEN signaling pathways [23]. The transcription factor PAX6 exerts a very strong neurogenic role. It has been proposed to be a regulator of balance between astrocytes and other glial cells [24]. PAX6 also interacts with neurogenins to swing the stem cells towards a neuronal commitment state.
  In a 2018 study, CRISPR-CAs9 was used to study the role of PAX6 in GBM development. PAX6 has a very strong neurogenic role, and its expression has been found to work as a tumor suppressor gene in GBM. The role of PAX6 is also pleiotropic in nature. Its signaling is involved in the differentiation process and is also capable of exerting impact on GBM oncogenesis [25]. Pax6 interacts with regulators of neural progenitor cells including Neurod1/4, Nestin, Neurog1/2, and Notch pathway components such as Dll1 and Hes6 [26].
  *It is very important to note that neuronal cells are the cell types with the least risk of tumor development while glial cells are most likely the cell types in which one of the most aggressive tumors originates, GBM. It is postulated here that there are many genes whose expression may provide a different effect in different cell types. This variability may have origin in cell fate specification based on the differentiation of cell types, such as PAX6 plays different roles in neurons and astrocytes. This is also evident in different disease states such as in Alzheimer’s disease and glioblastoma, in both diseases the genes such as p53 have different roles. In Alzheimer’s diseases, p53 is upregulated while in GBM, it is downregulated. PAX6 and Sox2 are involved in the gene regulation of neural stem cells in neurogenesis. Both are also contributors to the commitment of ectodermal cells towards neuronal differentiation. Their contributions also go to the extent of inducing transcription factors that contribute to neurogenesis. They work in the form of gene regulatory circuitry. PAX6 is deeply interlinked with notch signaling in inducing neurogenesis. It is not properly understood yet the level of complexity with which they work together. One of their key roles that are of immense significance is the repression of non-neuronal cell types.*
- **P300/CBP:** P300 is a very strong regulator of gliogenesis. It is also the repressor of nestin which is also involved with stemness (sox2). Its activation in GBM cell lines induces the cells more towards GFAP expression. The impact of P300 on the cell fate can be estimated from this fact. It is important to note here that the c-myc gene overrides p300 and then GFAP, leading to upregulation of nestin. This plays a very important role in GBM oncogenesis [27].
- **Neuronal restrictive silencing factor (NRSF)/REST:** It halts neurogenesis and induces gliogenesis. Initially, it was considered to function only to repress neuronal genes in non-neuronal cells. Now, its presence in many different cell types has emerged. It is expressed in neural progenitor cells (NPCs) but negatively regulates neurogenesis. It has also been found to be involved in many cancers [28]. It is considered to be contributory towards the neurodegeneration in Alzheimer’s disease as it is downregulated in Alzheimer’s disease neurons. It has been found that REST remains in a quiescent state in differentiated neurons, but its expression increases after neuronal injury. With aging, there is always present low level of REST gene expression in the neurons because its absence may fuel Alzheimer’s disease. REST also reduces beta-amyloid toxicity and apoptotic signaling [29]. In GBM, it contributes to more invasive and proliferative properties of GBM. It also contributes to the renewal of GBM stem cells and is oncogenic in GBM landscape [30].
  REST amplification is also implicated in GBM and has been found to suppress apoptosis in GBM cells. It also promotes stemness in GBM. Though NRSF/REST is considered to have a more profound role in gliogenesis, but in medulloblastoma where neuronal pathways are involved, its over-expression has been detected as it is linked to enhancing proliferative capabilities. REST-based stemness has been detected in both neuronal tumors such as medulloblastoma and glial tumors such as GBM. REST also interacts with shh, wnt, and PI3K signaling pathways. All of them have a well-established role in GBM oncogenesis. In adult neurons, the gene expression of REST remains low so as to promote the gene expression of neuron-specific genes which REST can downregulate. But REST is upregulated many folds in medulloblastoma cells, the role of REST overexpression is very important as blocking the REST gene expression results in the revival of neuronal genes and also promotes apoptosis [31]. REST overexpression is also part of GBM. It also provides a key finding about pro-neural type of GBM as when P53 and REST are deleted; it switches the cells to change into pro-neural type of GBM [32]. REST in normal cells contributes to genomic integrity, but in cancer development, it is oncogenic. It also contributes to invasion, stemness, and also regulates apoptosis [33]. NRSF/REST is highly upregulated in NSCs to maintain stemness and prevent neuronal differentiation. Its gene expression downregulation contributes to drive the neuronal differentiation. But after neuronal differentiation, a basal level of NRSF/REST expression is maintained all the life and increases with aging. There is aging-related increase in REST expression in brain cells including neurons and astrocytes. REST has strong interactions with WNT signaling pathways and both have significant oncogenic role in GBM. It also downregulates the genes involved in apoptosis [34].
  *The differences in REST gene expression are likely one of the key reasons of different proliferative potentials between two cell types, i.e., neurons and glial cells. It is postulated here that as REST is gliogenic and is not similarly expressed in neuronal cells which are considered permanent cells, hence the direction of cell circuitry guided by REST tends to have high proliferative potential. The REST-based proliferative potential is so strong that it is also involved in tumor development in neuronal cell type (medulloblastoma) by silencing the key neuronal cell type specific genes. NRSF/REST works as one complex developmental expression program. It has been found to be highly expressed in almost all non-neuronal cells. Hence, it is also considered to be a master regulator of transcription. One of its most significant roles is to regulate gene networks that are involved in maintaining pluripotency in embryonic stem cells.*
- **Leukemia inhibitor factor (LIF):** Normally, it inhibits differentiation but its low levels cause differentiation. In GBM, TGF-beta signaling causes it to become pro-oncogenic. Although when LIF is applied alone to cell lines, it causes growth inhibition in GBM [35].

#### Genes that have gliogenic roles but in GBM development contribute to the oncogenesis

IL-6 family, FGFR 3, JAK-STAT pathway, STAT3, S100, hey1, HES1, DTX, NF-κB, Neuregulin-1, MAPK, MEK, E2F, TCFL2, NFIX TF, Ephrins (EFNB1), and Netrins (NTN).

- **FGFR signaling pathway:** FGFR3 is one of the mitogenic drivers in GBM development. It also contributes to further driver mutations. In GBM disease development, it activates very crucial pathways such as AKT, Ras-Raf-MEK-ERK pathway [36]. FGFR signaling pathway is profoundly involved in cancers including brain cancer such as GBM [37]. In GBM, their role is complex and is based on their ability to contribute towards glial differentiation. FGFR signaling pathway has strong interactions with MAPK which is involved in gliogenesis and also in GBM development. FGFR2 is involved in gliogenic differentiation, and its activation in glioma cells causes differentiation of brain glioma cells [38]. FGFR 1 and 2 are expressed in neurons and astrocytes, respectively. Their expression decreases with grade of glioma [39–41]. It is important to mention here that IGF-1, FGF, PDGF, and EGF, all are based on receptor tyrosine kinase, and they all have significant roles in GBM development. FGF switches NSCs towards glial fate in embryogenesis. The cell fate-based switching is through MAPK pathway. FGF is also involved in GBM. FGF also works with IGF to regulate MAPK pathway. The role of IGF-1 in cancer development is well established. IGFR signaling pathway interacts with AKT to further GBM proliferation [42].
- **JAK-STAT pathway:** It is a key gliogenic pathway. It interacts with PI3K/AKT/mTOR Pathway. It has ability to integrate with MAPK/ERK pathway. There are negative regulators that keep JAK-STAT in check such as tumor suppressor genes. In GBM, JAK-STAT becomes oncogenic. It also contributes to the stem cell maintenance. The JAK-STAT pathway downstream targets include Bcl-xL, Bcl-2, cyclin D1, and c-Myc, and EGFR amplification which lead to further dysregulated STAT3 signaling. Dysregulated EGFR, FGF, PDGF, and c-MET also activate STAT-3 signaling. The EGFR-based negative regulatory mechanisms become dysregulated in GBM [43, 44]. The dysregulations in JAK/STAT signaling lead to upregulation of pluripotency related genes including oct4, c-Myc, Nanog, and Sox2 [45–49]. Sox2 is also involved with JAK-STAT signaling pathway in maintaining pluripotency. TGF Beta, SMAD, and IL6 family also interact significantly with JAK-STAT pathway [50–55]. Damage to RB2/p130 diminishes its tumor suppressor effect and contributes to GBM progression [56].
- **STAT3:** The role of JAK-STAT pathway and STAT3 is well-established in gliogenesis. Oncogenic mutations also dictate the role of STAT3 in relation to its interactions such as with PTEN-Akt-FOXO axis (suppressive) and with leukemia inhibitory factor receptor beta (oncogenic). It also interacts with EGFR which plays key role in GBM development [57, 58]. The knockdown of STAT3 or SRF significantly suppresses tumor invasive properties. It also interacts with HOPX which is gliogenic and also expresses tumor suppressive effects [59].
  *It is postulated here that genes and pathways including STAT3 have multiple biological roles which are also guided by the timing and gene expression of other factors that regulate the cell cycle. In tumor microenvironment, a physiologic pathway may go on to become oncogenic. Their integrated relationship with one another decides the maintenance of cell fate or deviation of cells from their cell fate.*
- ***IL-6 family:*** In GBM, it works with STAT3 in promoting pro-oncogenic pathways [60]. It is to note that cytokines IL-2, IL-6, and IFN, all involve JAK-STAT based non-receptor tyrosine kinase signaling. With age, the gene expression of inflammatory cytokines increase in the body. In GBM, CTNF and its receptor have been found in GBM [61].
  CTNFR alpha is pro-glioma and is linked to the grade of glioma. It is considered to have role in initiation or GBM maintenance [62, 63]. CNTF-mediated JAK-STAT pathway contributes to astrocytogenesis as well.
- ***G*FAP*and*S100:** They are involved in astrocytogenesis and are also upregulated in GBM, indicating the cell type-specific contributions to GBM development. GFAP also regulate astrocyte neuronal interactions [64].
- **Notch targets Hey1:** One of the key targets of Notch signaling is Hey1, and this is also upregulated in GBM. Notch signaling in later stages stimulates gliogenesis [65–68]. *It is postulated here the timings of gene activity and microenvironment impact profoundly in the process of maintaining cellular homeostasis and in disease development. The dysregulations in gene expression of Notch also induce profound impact on the genes it regulates and the pathological effect goes further in downstream pathways.*
- **Interactions of Hes1 with STAT3 and JAK2:** These interactions play very important role in gliogenic developmental mechanisms. But dysregulated expression of even the key genes including Notch1, Hes1, and DTX1 contributes to GBM pathogenesis [69–71]. Dysregulated Hes1 plays role in stemness and EMT induction in GBM development. Shh also upregulates Hes1 gene expression [72]. SHH will be discussed later.
  *In understanding GBM, it is of immense significance to understand interconnectedness of signaling pathways and genes. The interactions of Notch are very diverse as it also interacts with Hes3 signaling axis and STAT3 and plays physiologic role of being the regulator of neural progenitor cells (NPCs). In GBM, Notch, STAT3, and Hes3 axis all become dysregulated.*
- **NF-κB harms neural stem cells (NSCs) and gliogenesis potential:** The role of inflammation in GBM oncogenesis and its impact on the GBM genetic landscape is of immense significance. It is already well established that the levels of inflammation in body increases with aging. NF-κB which also acts as one of the prime regulators of inflammation, it gets upregulated. And NF-κB has been found to play very important role in GBM oncogenesis as it also plays role in EGFR amplification. The EGFR amplifications also contribute to the pyruvate kinase m2 (PKM2) dysregulations, resulting in the upregulation of this rate limiting enzyme of glycolysis in GBM. In GBM, NF-κB and EGFR interactions contribute to GBM development, invasiveness, and progression [73].
  There is also depletion of negative regulators of NF-κB such as KLF6. The upregulation of NF-κB in GBM also contributes to epithelial mesenchymal transition (EMT) and GBM stemness. The NF-κB is linked with TNF alpha and IL1 expression. It interacts with MAPK, tyrosine kinase-R, EFGR, PDGF, and AKt signaling pathways. They are also part of GBM landscape. The NF-κB signaling promotes IL-6, IL-8, and VEGF, further fueling GBM development. It also interacts with PI3K/AKT pathway. With loss of PTEN and NF1 which are well established contributors to GBM development, the PI3K/AKT becomes upregulated. Similarly like other tumor suppressors such as PTEN, NF1, and also loss of P53 contribute to GBM oncogenesis. The dysregulations in P53 also contribute to NF-κB upregulations. NF-κB also interacts with cascades of genes that are involved in cell survival including Bcl-xL*,* Bcl2*,* inhibitor of apoptosis proteins and survivin*,* and *c*yclin D1. The NF-κB in GBM has strong interactions with EFGR, PDGFR, and AKT signaling pathways. The loss of tumor suppressor in NF1 also causes RAS over-activation [74]. PTEN regulates PI3k and blocks AKT signaling. The dysregulations in P53 also contribute to Mdm amplifications in GBM oncogenesis. The NF-κB also disrupts the notch signaling and promotes the cell signaling through cell proliferation with CyclinD1 [75].
  *It is important to note here that NF-κB signaling upregulation is also involved with loss of tumor suppressors. It is also postulated here that inflammatory signaling cascades have more profound role in GBM oncogenesis than previously assumed. As aging also increases inflammation and most cases of GBM have onset with increased age, hence role of aging is of immense significance in GBM development.*
- **Neuregulin-1 is a gene of EGF family and contributes to astrocytogenesis:** Nrg1 and erb receptor signaling pathways interact with each other and also with PI3K, contributing to the growth of GBM. The role of EGFR amplification is already well established in GBM [76–79]. Nrg1, TGF alpha, and EGFR all have profound interactions with one another and impact PI3k/AKT pathway, MAPK, and JAK/STAT pathway [80].
  *It is postulated here that Nrg1 is very significant in GBM landscape as it is involved in astrocytogenesis during embryonic development but in GBM landscape, it becomes oncogenic. It signifies the role of cell fate-specific genetic programming that regulates the unique gene transcripts in a specific manner. In GBM, when dysregulations in signaling pathways and genes accumulate beyond a specific threshold, then the role of many factors such as Nrg1 may become dysregulated. This further fuels GBM development and progression. The FGFR3 has major gliogenic contributions, but in GBM development, it also loses the cell fate specification related effects. As the cell fate is determined by combinatorial code based specific unique set of gene programming, this loss of cell type-specific gene expression is able to swing FGFR3 towards becoming oncogenic.*
- **MAPK**: In GBM, it interacts with EGFR, mTOR/PI3K/Akt, and RAS. It also works with PDGFR. MAPK is also involved in insulin resistance. The pathway mTOR stimulates glucose uptake in GBM and works with Akt pathway [3].
- **MEK and E2F:** RAS over-expresses MEK in GBM. Rb1 interacts with CDKs in inhibiting E2F. This pathway becomes damaged in GBM and CDKs cause E2F based G1 to S transitions [81, 82].
- **TCFL2:** TCF/LEF transcription factors of WNT signaling works with beta catenin and have been detected in GBM samples [83–85].
- **Ephrins (EFNB1) and Netrins (NTN):** These pathway-related proteins are involved in gliogenesis. Their role also includes angiogenic activity including angiopoietin-2 (ANGPT2), EFNB1, and FGF. The dysregulations in Ephs and Ephrins are involved in GBM development. Their downstream signaling includes MAPK, ERK, RAS, AK, FGF, MEK, and PI3k/Akt/mTOR [86].
  Netrins over-expression contributes to the increased proliferation of GBM cell lines. It is also involved in gain of stemness in GBM. NTN-1 activates Notch signaling in GBM and contributes to oncogenesis. This increases the expression of CD133, nestin, and Sox2 in GBM stem cells [87]. Netrins also interact with EGFR. This reduces partially the GBM cell senescence that occurs from DNA damage. This EGFR-based role is mediated by AKT and ERK signaling pathways.
- **Transcription factors NFIX and HOPX with GFP control:** This is involved in gliogenesis and is dysregulated in GBM. NFIX TF interacts with STAT3 and has been found to be upregulated in GBM. NFIX works with Ezrin protein that is involved in cross-linking of cytoskeleton and plasma membrane; both are dysregulated in GBM [88]. HOPX is primarily astrocytogenic and also works as tumor suppressor in a time-dependent manner. HOPX is also involved in neural stem cells and is downregulated in solid tumors. This downregulation promotes tumor invasiveness and growth. It is important to note that HOPX over-expression is involved in suppression of cancer cell proliferation and metastasis. Key genes of interest with which HOPX interacts include ASCL2 and NKX2-1. HOPX contributes to regulation of NOTCH and also causes senescence by activating Ras and MAPK pathway. It is also involved in upregulating klf4 in promoting tissue homeostasis. Klf4 is involved in pluripotency and time-dependent switching of astrocytes into neurons. It also regulates apoptosis in stem cells. When it becomes dysfunctional, stem cell apoptosis decreases and more neurons are produced. Its upregulated gene expression promotes apoptosis. HOPX also contributes to neurogenesis by modulating NOTCH pathway signaling but it plays more profound role in gliogenesis [89].
  It is postulated here that role of HOPX is far different than other gliogenic factors that become oncogenic when dysregulated including Notch and FGFR3. Its effect on cancer cells is tumor-suppressive. It keeps NSCs in quiescent stage and also regulates proliferation and differentiation [90]. GBM lacks expression of HOPX and its induced expression causes tumor suppressor effect in many GBM cell lines by decreasing survival but proliferation remains unaffected [91].

***Key genes that contribute to gliogenesis but also contribute to the stemness in GBM:*** Notch (cancer stem cells), Sox9, Sox4, and SHH.

- **Notch signaling pathway:** In gliogenesis, Notch effector protein (NIFA) binds to GFAP promotor and contributes to astrocytogenesis. EGF motifs and notch signaling are interlinked. In GBM, EGFR amplifications are one of the most significant parts of GBM oncogenesis. GBM cells also have presence of cancer stem cells [92]. Notch2 expression is involved with proliferating cells that function as progenitors. Notch1 gene expression is involved in post-mitotic cells that are in differentiated state. In GBM, Notch1 promotes Akt signaling, also causes β-catenin and NF-κB upregulation. In GBM, Notch2 is also upregulated but contributes to GBM stemness. Notch signaling pathway also interacts with stemness genes (nestin*,* SOX2*),* with vimentin and GFAP that are involved in astrocyte fate. It also inhibits apoptosis [93]. Dlls are linked with Notch signaling. They are upregulated in GBM and contribute to oncogenesis [94–96]. Nestin upregulation contributes to GBM stem cells [97–99].
  Notch signaling is regulator of EGFR and EGFR amplifications are also modulated by dysregulations in TP53. Notch signaling and EGFR, both are upregulated in GBM oncogenesis. Importance of notch signaling in developmental mechanisms and its impact on GBM development both are very significant part of GBM research. They both work like positive feedback signaling loop. EGFR interacts with RAS-RAF-MEK-ERK and the PI3K-AKT-mTOR cascades. It also interacts with CDKs. Inhibition of notch also promotes apoptosis in GBM cells. Notch inhibition also decreases gene expression of Akt and Stat3. Delta/Notch-like epidermal growth factor-related receptor (DNER) is involved in GBM progression. The notch signaling pathway also regulates neural stem cells (NSCs). Though Notch is also involved in cell fate decisions in gliogenesis but in GBM microenvironment, it makes tumor cells more invasive and increases de-differentiation. Higher grade of gliomas have been linked with increased gene expression of ASCL1, Dll1, notch1, notch3, notch4, and hey1. The ASCL1 is also used as a transcription factor in the switching of astrocytes to neurons. Dysregulated Notch also interacts with other important developmental genes and gene expression levels of Notch1, Notch4, Dll1, Dll4, Jagged1, CBF1, Hey1, Hey2, and Hes1 are upregulated in GBM oncogenesis. There is also simultaneous decrease in PTEN expression, resulting in upregulation of Akt and VEGF. The notch mediates overexpression of genes including Zeb1, and Snail, vimentin. This enhances invasiveness of GBM. The notch signaling also interacts with Hey1, PI3K/AKT/mTOR, and ERK/MAPK pathway. All of them also act as proliferative and survival signaling [100]. Hey1 enhances GBM survival and interacts with dysregulated Notch and E2F signaling. It is considered that the notch signaling is one of those key pathways whose inhibition can promote GBM cells to differentiate and promote apoptosis. Despite having such crucial role, still inhibition of notch signaling pathway does not reverse GBM oncogenesis [101]. Notch signaling is an important player in gliogenesis. The oncogenic alterations in notch signaling pathway sets in motion a cascade of pathological downstream dysregulation such as hey1 gene which interacts and works with notch signaling, and its gene expression is increased in GBM development [65].
  *NOTCH works with FGF to keep NSCs in proliferative stage. Neural progenitor cells (NPCs) are regulated by notch signaling, and it is also involved in their maintenance and renewal. But later in developmental, NOTCH contributes to glial cell differentiation*.
- **SOX9 and SOX4:** It is involved in gliogenesis. The pathways Shh and Notch use Sox9 as regulator of morphogenesis. It also works with SOX10 for maintenance multi-potency of neural crest cells. It also differentiates NSCs into non-neuronal cells. Sox9 upregulation contributes to oncogenesis in GBM and interacts with wnt/beta catenin for progression of GBM. Knockdown of Sox9 in GBM impairs the proliferation of glial cell types and cause apoptosis [102, 103]. Sox9 interacts with EGFR, BMI-1, and PTEN. Other SOX including Sox5, Sox6, and Sox17 also contribute to GBM. SOX4 in Glioblastoma sustains stemness and is regulated by TGF-β. It also modulates SOX2. The SOX2 in glioblastoma maintains stemness and oncogenic properties. Sox members in GBM also interact with TGF beta and are involved in reprogramming of GBM stem cells [104].
  *It is postulated here that factors that swing the stem cells towards non-neuronal cell types which are not permanent in nature and have proliferative capacity, they are at risk of oncogenesis later in life. Many genes such as Sox9 that are gliogenic in embryonic development may become contributor to GBM oncogenesis because of dysregulations in cell circuitry at the time of GBM development. Such dysregulations change the direction of cell fate, and they become more and more undifferentiated. It is important to note that sox9 and NIFA together work as regulator of key gliogenic genes in embryonic development. Their over-expression cause astrocytic differentiation in GBM cells. Many pathways that work in a combinatorial manner may get dysregulated with aging. Such dysregulations in cell circuitry lead to disease development such as GBM. The transcription factor NIFA, key initiator of gliogenesis works with Notch and Hes signaling pathway, and pathological dysregulations in one key pathway lead to further downstream damage. It is further discussed in later parts of the study.*
- **SHH:** Astrocytogenesis is linked with inhibition of neurogenic signals in neural stem cells (NSCs). While SHH is secreted by neurons and work more as a connecting bridge between neurons and glial cell. It works with Nkx2.2 in defining the identity of neural progenitor cells. But still it also contributes to the development of glial cells as well [105]. Sox9 is downstream effector of Notch and SHH. The role of SHH in GBM becomes more significant as Hes1 that is also involved in stemness of GBM and is also upregulated by SHH [106–108].
  The role of PTEN is very significant as it also contributes towards controlling the SHH and PI3K expression. When PTEN becomes defective in GBM, then SHH and PI3K become dysregulated and both contribute to GBM oncogenesis [109]. SHH works as a morphogen but its sensitivity in neural cells is determined by BMPs. SHH have more profound role in neurogenesis compared to gliogenesis. SHH regulates and induces a variety of regulatory genes including Nkx2.2, Olig2, Nkx6.1, Nkx6.2, Dbx1, Dbx2, Irx3, Pax6, and Pax7. This is mentioned here to show the far-reaching impact of SHH dysregulation in GBM development. Cross-talk of SHH with many key pathways and genes plays major role in regulating the activity of all of them. Such major interactions include GSK3 beta, mitogen-activated protein kinase (Mek1), PKA and protein kinase C (PKC), Phosphoinositide-3 kinase (PI3K), CK1, or dual specificity Yak1-related kinase (DYRK1). SHH is also modulated by these interactions. SHH also has major interactions with key signaling pathways including TGF-beta, wnt/beta catenin, and notch. It also interacts with K-RAS, PKA, and EGFR. The signaling pathway SHH has profound regulatory interactions with GSK3 beta as it can act both as negative regulator and positive regulator of SHH depending on the microenvironment. Many major signaling pathways and key genes have regulatory interactions with shh, notch, wnt signaling, ERK signaling pathway, wnt/β-catenin and KRAS, TGF-β/TGF-βR, EGFR, and platelet-derived growth factor receptor α (PDGFRα). In GBM development, their dysregulation plays a key role in progression of the oncogenesis [110, 111].
  EGFR that is considered to play key role in GBM development, its cross-talk with SHH in GBM further fuels oncogenesis. SHH dysregulations are capable of inducing the profound impact on the gene expression of EGFR/RAS/RAF/MEK/ERK in different cancer cell lines [112]. The EGFR signaling downregulates the gene expression of negative regulators of Shh signaling, further fueling cascade of downstream changes by impacting the gene expression of Akt, ERK, and others [106].
  *It is postulated here that as cell fate is determined by unique combinations of transcription factors and regulatory genes. When such key genes and signaling pathways including SHH become dysregulated, the cells switch towards de-differentiation. This downstream cascade of damage works like a positive feedback loop that keeps switching the cells towards higher grade and more aggressiveness. There exists a very delicate balance among the expression of different genes which exert their effect on one another. For example, PTEN acts as a tumor suppressor and it also impacts SHH and PI3K. When PTEN is damaged in GBM development, then it produces number of downstream pathological changes. The role of SHH in inducing and determining the cell fate is concentration and time dependent. The dysregulations in signaling pathways such as SHH in GBM landscape and development can set in motion a cascade of oncogenic changes. This cascade is based on the interactions of genes involved and their roles in cell circuitry. The negative and positive regulatory effects of genes and signaling pathways are of major significance as dysregulations in one can set in motion a cascade of pathologic changes. Such changes unfold step by step and in time-dependent manner influenced by their microenvironment.*

*Related neurogenic genes/signaling pathways and their role in GBM oncogenesis:* GBM cells maintain low or null expression of neurogenic genes. Here, we investigate their significance in GBM [113].

*Key neurogenic genes having the ability to control oncogenesis in glioblastoma cells:* PAX6, neurogenins including Ngn1, NeuroD1, NeuroD4, Numb, NKX6-1 Ebf, Myt1, and ASCL1.

- **Neurogenins including Ngn1:** It prevents the interaction between p300/CBP complex and STAT3, thus preventing gliogenesis and promoting neuronal differentiation. In GBM, its expression causes mitotic arrest [114, 115].
- **NeuroD:** It is also the most potent key regulator of neurogenesis and is involved in the differentiation of neuron. In GBM, its induced gene expression also blocks the proliferation. NeuroD1 is so potent that it is able to switch reactive glial cells into neurons. Key interactions include Cyclin D1, MAFA, and MAP 3K10. The NeuroD1 and NeuroD4 are key neurogenic pathways. Their upregulation cause arrest of cell cycle in GBM cells.
  But the BDNF works in close association with NeuroD and activates the neurogenic SHP2-Ras-Raf-MEK-ERK pathway. This interferes with gliogenic JAK-STAT signaling. The neurogenic SHP2 is linked with potentiation of MEK-ERK signaling and inhibits gliogenic JAK-STAT signaling. In GBM, SHP2 becomes dysregulated and oncogenic [116].
- **BMP in neuronal development:** It is involved primarily in the induction of neurogenin and ASCL1. The BMPS are involved in both gliogenesis and neurogenesis, but play a major role in switching NSCs towards astrocytogenesis. And its expression is also present postnatally in astrocytes. BMP upregulation is considered to play role in halting GBM progression [14, 15].
- **PAX6**: It is neurogenic, but in GBM development, its expression is like a tumor suppressor. It interacts with neurogenins to swing the neural stem cells towards neural commitment state and is involved in the repression of non-neuronal cell types. When weak Shh signaling combines with strong TGF-beta signaling, the PAX6 becomes upregulated. PAX6 expression is maintained in many areas of brain throughout life [19, 20, 22].
- **Numb gene negates Notch signaling and contributes to neuronal differentiation**: Upregulation of Numb gene contributes to halting the GBM growth and progression. It also upregulates the Pax6 and Sox2. It also interacts with EGFR. But the functions of Numb are so diverse and complex as it has also been found to be highly upregulated in mesenchymal GBM cells [117]. But this may be due to the fact that despite gene expression upregulation GBM oncogenesis is beyond the control of Numb. But still its tumor suppressor role is controversial [118].
- **Nkx6.1***:* Increasing evidence indicates that it suppresses tumor development and metastasis [119, 120].
- **ASCL1**: It is strongly neurogenic. In GBM, its expression causes cascade of downstream changes and switch the cells towards neuronal cell fate. It also suppresses oncogenesis in GBM cells [121].
- **Ebf:** its loss in GBM contributes to oncogenesis because EBF3 downregulates the gene expression of proliferation and survival related genes including cyclins, CDKs, Mcl-1, and Daxx. It also upregulates genes involved in cell cycle arrest including p21 and p27 [122].
- **Myt1:** It is neurogenic transcriptions factor. In GBM, its expression is involved in downregulating proliferation [123].

*Genes that have neurogenic roles but in GBM development contribute to the oncogenesis:* PDGF, NT3, Pax7, Dbx2, hes6, Runx1, and Runx2.

- **PDGF and NT3:** Both are neurogenic, but in GBM landscape, they play oncogenic role [124]. PDGFR expression is increased in all grades of glioma and NT3 is upregulated in GBM development.
- **CNS neural progenitor markers:** Pax7 and Dbx2. Pax7 becomes upregulated in GBM with PTEN deficiency [125] and high DBX2 in GBM is linked with low survival [126]. Pax7 is involved in GBM invasiveness and oncogenic transformation of NSCs. Dbx2 works with REST in GBM proliferation.
- **Hes6:** Upregulated in GBM [71]. Key interactions with p53, NF-κB, and c-myc genes. It is involved in angiogenesis, proliferation, and migration in GBM oncogenesis.
- **Runx1 and Runx2**: In GBM, there role is oncogenic [127, 128]. Some studies have suggested that they may have oncosuppressive roles in the early part of GBM development, but it is still controversial.

*Key genes that contribute to neurogenesis but also contribute to the stemness in GBM:* klf4, myc, oct2, sox4, TLF TF, wnt signaling, sox2, sox4, Notch/STAT3-Ser/Hes3 Axis, and GSK3beta also work with PI3K/FGF signaling pathway and contribute to stability of c-myc.

- **Wnt signaling:** It is kept in check by tumor suppressors and is more involved in neurogenesis. It is also involved in insulin-based increase in glucose transporters. This increase is mediated by activation of wnt/beta catenin signaling pathway. It also plays role in diabetes type2 pathogenesis. In neurogenesis, wnt acts on Pax6-Ngn2-Tbr2-NeuroD-Tbr1 based neurogenesis TFs cascade. The wnt signaling represses astrogliogenesis via ngn2-dependent direct suppression of astrocyte gene expression. When wnt is inhibited, this promotes gliogenesis. Wnt1 works as antagonist of neural differentiation and promotes the proliferation of neural stem cells (NSCs). But in GBM landscape, it becomes highly oncogenic. Cancer stem cells are heavily associated with WNT signaling. Dysregulated wnt signaling causes activation of CyclinD1 and c-myc, causing G1 to S phase transition. It also contributes to epithelial to mesenchymal transitions [129–131].
- **GSK3:** It has already been discussed in other sections too. Here, we point out the integrated nature of GSK3beta interactions in normal cells and cancer cells. It also focuses on interconnectedness of beta catenin and GSK3-beta in development of GBM. The GSK3 and more specifically GSK3beta play a very significant role in normal cells and cancer cells. In normal cells, it acts as negative regulator of epithelial-mesenchymal-transitions (EMTs) and many proto-oncogenes. But dysregulated GSK3beta is oncogenic [132]. Even in Alzheimer’s diseases, it plays a very important role by forming a complex with p53 and contributes to neurodegeneration [133]. In developmental biology, GSK3 deletions contribute towards the enhanced proliferations of neural progenitor cells with simultaneous increase in SOX2 and beta-catenin expression. The GSK3 activation is involved in neuronal differentiation [134]. GSK3-beta has important interactions with beta-catenin which is capable of inhibiting neuronal differentiation. It also works with PI3K/FGF signaling pathway and contributes to the stability of c-Myc. In GBM, all of them become dysregulated [135]. In GBM, dysregulated GSK3-beta also acts to downregulate BMP that has significant gliogenic roles. It also downregulates CDKN1A and other important genes. GSK3 has interactions with many genes and pathways that are involved in GBM such as PI3k/AKT/mTOR pathway, wnt, notch, shh, ras, raf, mek, erk, APC, and axin. It also interacts with key tumor suppressors including p53 and PTEN [136, 137]. Though its main role is with neural progenitor cells and neurogenesis, but it is estimated that it may also have contributions towards gliogenesis [138]. *There may be a possible relationship between GBM and diabetes type2. The GSK3 plays significant role in both the diseases. Inactivation of GSK3-Beta contributes to the insulin resistance. As astrocytes are glycogen storing cells and GSK3beta also has major association with PI3k/AKT pathway in diabetes type 2 and in GBM, this should be evaluated in great detail in future studies. This may point to some possible significance or relationship between diabetes type2 and GBM as the risk of both increases exponentially with age. Similarly, wnt signaling dysregulations have also been associated with diabetes type2. The MAPK, PI3k, AKT, mTOR, and many other common genes/signaling pathways that are involved in disease development mechanisms of both GBM and Diabetes type 2, and they should be investigated for any possible profound relationship. Investigating it any further is beyond the scope of this study.*
- **Myc**: It is also involved in neurogenesis but is oncogenic in GBM development [139]. It is important to note here that myc gene overrides p300 and then GFAP, leading to upregulation of nestin. This plays very important role in GBM oncogenesis [27]. GSK3beta also works with PI3K/FGF signaling pathway and contributes to stability of c-Myc.
- **Pluripotency in NSCs and brief look on their role in GBM: SOX2 and SOX4.** Sox2 is associated with regulation and maintenance of neural stem cells. It becomes downregulated during later stages of differentiation. The role of Sox2 in neurogenesis is very important because Sox2 also regulates the gene expression of other key genes. SOX2 induces ASCL1 and TLX TF. It alone is a very strong switch to neurons from astrocytes. In GBM, stemness is mediated by SOX2 and SOX4. They are also modulated by TGF-beta [104]. It also has several important key interactions with LIF signaling and Klf4. The dysregulations in both of them are also involved in development of GBM stem cells and invasiveness. The role of wnt signaling has already been explored in earlier sections of this study. Nanog, oct4, and myc are also major contributors to GBM stemness [140].
- **Klf4:** Neurogenic but acts like an oncogene in GBM [141]. It contributes to neurogenesis and pluripotency in NSCs. Klf4 is involved in GBM heterogeneity and GBM stem cell development.
- **SOX2 induces TLX TF:** TLX transcription factor works like an oncogene in GBM. It inhibits TGF-beta that causes cytostasis, and this leads to the progression of oncogenesis [142, 143].
- **Oct4**: It is involved in pluripotency and stemness. Its expression is several fold upregulated in GBM. The FGF signaling pathway is involved in neuronal cell fate determination despite presence of oct-4 gene expression. Without FGF signaling, the neural progenitor cell can revert back to embryonic stem cell like state with predominant oct-4 expression [144–149].
- **Notch/STAT3-Ser/Hes3 Axis:** This is neurogenic axis, but it is linked to cancer development and diabetes. In GBM, it impacts the cascades of downstream signaling pathways. This signaling axis is important regulator of NSCs. Its major activators include insulin, Tie2 (involved in angiogenesis), and Notch. The upregulation of this axis has the ability to oppose the development of neurodegenerative diseases [92, 93, 150–153]. *Hes3 has role of pro-survival signaling. It works with a strong relationship to SHH and potentiates FGF and EGFR signaling. It is one of the key regulators with ability to reprogram cells into NSCs. In GBM, this signaling axis becomes severely dysregulated.*

### Summary: Gliogenic genes/signaling pathways and their role in glioblastoma development

- Key gliogenic genes having the ability to control oncogenesis in glioblastoma cells: p300, BMP, PAX6 (Anti-GBM override), HOPX (tumor suppressive + differentiation), NRSF/REST (astrocytogenic but capable of playing oncogenic role), LIF, and TGF beta.
- Genes that have gliogenic roles but in glioblastoma development contribute to the oncogenesis: IL-6, FGFR 3, JAK-STAT pathway, STAT3, S100, hey1, HES1, and DTX, NF-κB, Neuregulin-1, MAPK, MEK, E2F, TCFL2, NFIX TF, Ephrins (EFNB1), and Netrins (NTN)
- Key genes that contribute to gliogenesis but also contribute to stemness in GBM: Notch (cancer stem cells), Sox9, Sox4, and SHH. Note: Other stemness-related genes such as nanog, oct4, FGF2, and others also get upregulated in GBM and contribute to the stemness in glioblastoma oncogenesis. But the prime focus of this study is to focus on key gliogenic and neurogenic genes/signaling pathways that are involved in cell fate maintenance and differentiation. This study focuses on investigating their role in GBM development and progression.

### Related neurogenic genes/signaling pathways and their role in GBM oncogenesis

- Key neurogenic genes having the ability to control oncogenesis in glioblastoma cells: PAX6, neurogenins including Ngn1, NeuroD1, NeuroD4, Numb, NKX6-1 Ebf, Myt1, and ASCL1.
- Genes that have neurogenic roles but in GBM development contribute to the process of oncogenesis: PDGF, NT3, Pax7, Dbx2, hes6, Runx1, and Runx2.
- Key genes that contribute to neurogenesis but also contribute to the stemness in GBM: klf4, c-myc, oct2/4, TLF TF, wnt signaling, sox2, sox4, and Notch/STAT3-Ser/Hes3 Axis; GSK3-beta works with PI3K/FGF signaling pathway and contributes to the stability of c-Myc.

### Glioblastoma core pathway abnormalities

Furthermore, glioblastomas are broadly divided into the following core pathway abnormalities. They are focused here to a limited extent because further focus is beyond the scope of this study.

1. RTK/RAS/PI3K signal alteration
  **Receptor tyrosine kinase in GBM and gliogenesis/neurogenesis**
    The most significant RTKs involved in glioblastoma include EGFR, IGFR, PDGFR and VEGFR [154].
  **EGFR:** Its amplification is considered to play a key role in GBM development. In gliogenesis, JAK-STAT pathway interacts with EGFR. Dysregulated EGFR also activates STAT3 signaling. Both are considered to be important players in the glioblastoma development [43, 44]. The negative regulatory mechanisms of EGFR become dysregulated in GBM [92].
    BMPs act as a gliogenic regulator in the process of gliogenesis. They act as a negative regulator of EGFR. It is important to remember that BMPs cause the cell cycle to exit [12, 13]. Notch signaling is involved in astrocytogenesis. The EGFR is also interlinked with NOTCH signaling. Notch signaling is regulator of EGFR, and this EGFR amplification is also modulated by dysregulations in TP53. Both the notch signaling and EGFR are upregulated in GBM. EGFR interacts with RAS-RAF-MEK-ERK and the PI3K-AKT-mTOR cascades. It also interacts with CDKs. SOX9 is involved in gliogenesis, and its dysregulation contributes to stemness in GBM. SOX9 interacts with EGFR in GBM oncogenesis [104]. EGFR-AKT-Smad signaling is also another way via which SMAD contributes to GBM [17].
    EGFR is considered to play key role in GBM development. Its cross-talk with SHH in GBM further fuels oncogenesis. The EGFR signaling downregulates the gene expression of negative regulators of Shh signaling pathway. This further fuels the cascade of downstream pathological changes such as by impacting the gene expression of Akt, ERK, and others [57, 58].
    NF-κB has been found to play a very important role in GBM oncogenesis as it also plays role in EGFR amplification. EGFR amplifications also contribute to pyruvate kinase M2 (PKM2) dysregulations, resulting in the upregulation of this rate limiting enzyme of glycolysis in GBM. In GBM, NF-κB and EGFR interactions contribute to GBM oncogenesis. In GBM, EGFR amplifications impact the MAPK, PI3k/AKT pathway, and JAK/STAT pathway and contribute to the progression of GBM development.
    Netrins play a very important role in gliogenesis, and their role is also crucial in GBM oncogenesis. Netrins also interact with the EGFR. This reduces partially the GBM cell senescence that occurs from DNA damage. This EGFR based role is mediated by AKT and ERK signaling pathway [110, 111].
    In neurogenesis, EGFR interacts with the Numb gene to modulate the process of neuronal differentiation. Numb gene negates Notch signaling and contributes to neuronal differentiation [117]. Upregulation of Numb genes contributes to halting GBM growth and progression [76–79, 112].
    Another very important role of EGFR in neurogenesis includes its involvement with Notch/STAT3-Ser/Hes3 Axis. Hes3 has role of pro-survival signaling. It has strong relationship with SHH and potentiates the FGF and EGFR signaling. It is one of the key regulators with the ability to reprogram cells into NSCs. In GBM, this signaling axis becomes severely dysregulated [106].
  **PDGFR:** It plays a diverse role in the gliogenesis and neurogenesis. It plays a very important role in neurogenesis, but in the process of GBM development, it contributes to oncogenesis. Both PDGF and NT3 are neurogenic in neuronal development, but in GBM landscape, they play an oncogenic role [110, 111].
    It is important to mention here that IGF-1, FGF, PDGF, EGF, and insulin are based on receptor tyrosine kinase, and they all have significant roles in GBM development. Like EGFR, dysregulated PDGFR also activate STAT3 signaling [74]. The SMAD proteins which are involved in gliogenesis are of key significance. Dysregulations in SMAD alter the role of PDGF-B in GBM. When Smad, PI3K, and FoxG1 signaling pathways are dysregulated, they switch the TGF-beta to become oncogenic.
    In GBM development, when SHH becomes deregulated, it also contributes to dysregulated PDGFR signaling. NF-κB is linked with TNF alpha and IL1 expression. It has major interaction with PDGFR in GBM oncogenesis. In gliogenesis, PDGFR works with the MAPK pathway. When PDGFR becomes dysregulated in GBM, it also impacts the MAPK signaling [75].
    **IGF-1:** It works with FGFR3 in gliogenesis, but it becomes one of the key oncogenic drivers in GBM development. FGF also works with IGF-1 to regulate the MAPK pathway. The role of IGF-1 in cancer development is well established. IGFR signaling pathway interacts with AKT to further the GBM proliferation [39–42].
    **VEGFR:** When Notch signaling that plays key role in gliogenesis becomes dysregulated, this results in upregulation of Akt and VEGF signaling and contribute to GBM development. There is also simultaneous decrease in PTEN expression [18]. PAX6 and TGF beta also control the VEGF expression in GBM and prevents angiogenesis. They both stop the cells from entering S-phase of cell cycle and also induce apoptosis. The NF-κB promotes IL6, IL8, and VEGF, and this further fuels the GBM development [23, 74].
  **RAS pathway in GBM and gliogenesis/neurogenesis:** It becomes affected by alterations in FGFR3 which is one of the key gliogenic pathways. The alterations in Notch and SHH also impact the EGFR which in turn also impacts the RAS signaling. The loss of tumor suppressor in NF1 also causes RAS over-activation. Dysregulations in RAS also contribute to MAPK signaling alterations and contribute to GBM oncogenesis [36, 74, 106].
    MEK signaling is involved in gliogenesis. The RAS over-expression also dysregulates the MEK signaling in GBM. HOPX contributes to the regulation of NOTCH signaling and also causes senescence by activating the Ras and MAPK pathway [110, 111].
    Ephrins which are involved in gliogenesis. In GBM, the dysregulation of ephrins contribute to the RAS over-activation [86, 89]. In neuronal differentiation, RAS interacts with key neuronal differentiation factor NeuroD. GSK3 beta has important contributions towards GBM oncogenesis. It also contributes to the GBM progression by contributing to RAS over-expression [136, 137].
  **PI3K/AKT/mTOR**: JAK-STAT pathway, MAPK, EGFR, and NOTCH have major interactions with PI3K/AKT/mTOR Pathway in process of gliogenesis [43, 44]. SMAD dysregulations also impact PI3K/AKT/mTOR and cause the TGF beta to become oncogenic [16]. EGFR-AKT-Smad signaling is also another way through which SMAD contributes to the GBM. The TGF-β1 works with Smad, p38 MAPK, and PI3K/Akt signaling pathways in GBM development [17]. Neuregulin-1 is a gene of EGF family and contributes to the astrocytogenesis. Nrg1 and erb receptor signaling pathway interact with each other and also with PI3K pathway, thus contributing to the growth of GBM [76–79].
    NRSF/REST halts the neurogenesis and induces gliogenesis. It works with PI3k in GBM oncogenesis [28]. The GSK3 also works with PI3K/FGF signaling pathway and contributes to the stability of c-Myc. In GBM, all of them become dysregulated. GSK3 has interactions with many genes and pathways that are involved in GBM such as PI3k/AKT/mTOR pathway [136, 137]. PI3k/AKT/mTOR pathway contributes to the gliogenesis and neurogenesis but also contributes to stemness in GBM [138].
  **PTEN:** The role of PTEN is very significant as it also contributes towards controlling the SHH and PI3K expression. When PTEN becomes defective in GBM, then SHH and PI3K becomes dysregulated and both contribute to the oncogenesis [100, 109]. PTEN acts as a tumor suppressor and it also impacts SHH and PI3K pathways.
    NF-κB also interacts with the PI3K/AKT pathway. With loss of PTEN and NF1, the PI3K/AKT pathway also becomes dysregulated [74, 104]. PTEN regulates PI3k and blocks AKT signaling [75].
    Notch dysregulation causes simultaneous decrease in PTEN expression, resulting in the upregulation of Akt and VEGF. Sox9 which is also involved in maintaining pluripotency of NSCs also interacts with PTEN. The PTEN works as a tumor suppressor by controlling the gene expression of STAT3 [57, 58].
    PAX6 stops the cells from entering S-phase of cell cycle and also induces apoptosis. It also interacts with PTEN to regulate the cell cycle [23]. Loss of PTEN causes the PI3k/Akt pathway to become dysregulated. CNS neural progenitor marker Pax7 also becomes upregulated in glioblastoma with PTEN deficiency [125]. GSK3 beta also interacts with tumor suppressors including PTEN [136, 137].
  **NF1:** NF1 contributes to the regulation of NSCs proliferation and gliogenesis. Loss of NF1 leads to the drastic effects that lead to dysregulated NSC proliferation and uncontrolled gliogenesis. This also impacts the downstream signaling, and most significantly, PI3K/Akt/mTOR pathway becomes dysregulated as a consequence. The NF1 negatively regulates the RAS-signaling pathway. With loss of NF1, the RAS/MAPK signaling pathways become dysregulated [155, 156].
    Defects in NF1 impact both neuronal and glial cells. The impact of NF1 loss on neuronal cells is also of key significance as it contributed to abnormalities in cAMP generation in such cells. The GFAP works also by interacting with NF1 and it has capacity to impact the NF1 gene expression in order to enhance glial proliferation. The NF1 expression is essential in neuronal differentiation as well. Other key pathways that interact with NF1 include Notch, ERK, MEK, SMAD3, and Hes1 [157].
    ERK pathway works in a delicate regulated balance with NF1. The ERK pathway works as a G1 to S transition switch when upregulated. It has key interactions with many key regulator genes/signaling pathways such as SHH, FGFR3, JAK-STAT, Notch, EGFR, ephrins, netrins, NeuroD, GSK3, and CDKs [158, 159]. Nf1 inactivation leads to the increased gliogenesis, and this declines the neurogenesis. The MEK/ERK pathway becomes upregulated and hyperactive. This dysregulates NSC proliferation and increases the risk of GBM [160]. Nf1 mutations are of prime significance in mesenchymal subtype of GBM [161].
  **FOXO axis:** This axis promotes differentiation and negatively regulates proliferation. This axis is dysregulated in GBM. The PI3k/AKT pathway negatively regulates FOXO axis. This axis interacts with IGF1 as well. The role of FOXO is also governed by the pathways with which it interacts. In GBM oncogenesis, the dysregulated Akt cause the FOXO axis to become dysfunctional [162]. FOXO axis plays very significant roles as it works with TGF beta/SMAD axis. This plays a vital role in the process of gliogenesis and neurogenesis. The IGF1 suppresses FOXO axis. IGF1 signaling also promotes cdkn1a expression [163].
    PI3k/Akt, RAS, and MAPK pathways inhibit the FOXO axis. They all have well established role in GBM development [164]. FOXO axis is damaged in GBM. The physiologic role of FOXO is also to regulate cell cycle. It also enhances the expression of sirtuin1 during cellular senescence. Several studies have indicated that FOXO1 has the ability to inhibit EMT and metastasis [165]. FOXO is also being considered a target in treating GBM [166, 167]. In GBM oncogenesis, Foxo/SMAD signaling is downregulated while FoxG1 works to enhance proliferation by increasing the expression of Sox2 and Sox5 [168].
2. P53 signal alteration
  **P53:** P53 plays diverse functions ranging from neurogenesis to NSCs and brain development. P53 is dysregulated in GBM oncogenesis. The consequences of p53 loss on neuronal differentiation are still controversial as it is yet to be fully understood how it impacts NSCs. Although it is likely that p53 inhibits neuronal differentiation of NSCs [169, 170].
    Studies involving brain organoids have shown p53 to be a crucial player in CNS development. It also contributes to genomic stability and regulates neurogenesis [171]. Dysregulations in P53 also contribute to Mdm amplifications in GBM. In GBM, damage to P53 also dysregulates the notch signaling, GSK3 beta, Ras/MAPK, PI3k/mTOR pathway, and EGFR signaling and others.
    It is important to note that deletions of P53 and REST are so significant that it causes the cells to switch into pro-neural type of GBM. Loss of p53 in Li-Fraumeni syndrome also results in GBM development. This also makes p53 a major player in GBM oncogenesis [172, 173].
    **Cyclin-dependent kinase inhibitor 2A (CDKN2A):** It is a tumor suppressor and works with Rb1 to halt G1 to S phase transition. It encodes two tumor suppressor proteins P16 and ARF. CDKN2A mutations are associated with Glioblastoma development. ARF functions as stabilizer of P53 and sequesters the MDM2 which is a degrader of p53. CDKN2A mutation is also linked with EGFR amplification that is a major part of GBM oncogenesis [174]. MDM2 and MDM4 both have structural similarities and are p53 binding proteins. MDM4 is capable of reversing MDM2-based degradation of p53, but the apoptotic functions of p53 remain suppressed. MDM2 becomes over-expressed in GBM resulting in increased degradation of p53 [175]. Aging is also considered to play important role in the dysregulation of EGFR, Mdm2, and p53 [176].
3. RB signal alteration:
  **RB1:** Rb1 is a tumor suppressor. It is also involved in neuronal differentiation. Rb1 interacts with CDKs in regulating cell cycle. This pathway becomes damaged in GBM and CDKs cause E2F-based G1 to S transition [177]. It also contributes to differentiate the NSCs into astrocytes. Mutated Rb1 is considered a major player in GBM oncogenesis. P16-CDK4-RB pathway works in an interconnected manner and control cell cycle progression [178]. Rb1 knockout mouse models have been shown to increase the neurogenesis as a result. Rb1 interacts with CDKs in inhibiting E2F. This pathway becomes damaged in GBM and CDKs cause E2F based G1 to S transition [179].
    **Cyclin-dependent kinases and inhibitors:** CDKN2B forms complex with CDK4 and CDK6. This halts the activation of CDKs. CDKN2B regulates the progression of G1 phase of cell cycle. CDKN2C also forms the same complex, but it works more with Rb1 expression. TGF-beta induces CDKN2B. It regulates the cell cycle and is also key player in gliogenesis. TGF beta works with SMAD and CDKN2B to regulate cell cycle and gliogenesis. They also inhibit c-myc. CDKN2B also plays role in neurogenesis while working with PAX6. It is important to mention here that Sox2 and sox4 are involved in the stemness of GBM cells, they cause the downregulation of CDKN2B [180, 181].
    CCND2 forms complex with CDK4 and CDK6. This contributes to G1 to S cell transitions and inactivates the Rb1 for this purpose [182].

**Some other mutations in GBM:** briefly explored here as deeper investigation into all of them is beyond the scope of this study.

- **IDH1:** Mutant IDH1 contributes to the GBM development and ultimately contributes to oncogenesis through epigenetic mechanisms as well [183]. IDH1 mutation alters the neurogenic niche and promotes glioma formation. Resultant accumulation of 2-hydroxyglutarate alters DNA methylation and histone binding. This contributes to the oncogenic changes in neuronal and glial cell types [184]. Similarly, IDH2 has also emerged to play similar role in GBM oncogenesis.
- IDH mutations are considered to be among the initial mutations in GBM development. They are also common in diffuse gliomas. IDH mutations are more common in secondary GBM and far less common in primary GBM [185]. 1p/19q loss has been found to occur with IDH1 mutations in oligodendrogliomas [186].
- **TERT:** It is upregulated in stem cells including cancer stem cells. It is also related to the pluripotency of cells. It interacts with STAT3 which also has major involvement in the process of gliogenesis. The dysregulated STAT3 signaling is involved in glioblastoma development. Another major interaction of TERT is with GSK3 beta. The GSK3-beta deletions contribute to the enhanced proliferations of neural progenitor cells with simultaneous increase in SOX2 and beta-catenin. In normal cells, it acts as negative regulator of epithelial-mesenchymal-transitions (EMTs) and many proto-oncogenes. But dysregulated GSK3-beta is oncogenic. TERT plays a wide array of roles ranging from cellular aging to epigenetic clock [187, 188].
- Similarly, ATRX is important component of chromatin remodeling complex. Its dysfunction also causes immense genomic instability. This alteration is present in 44% of GBM [189].
- **FUBP1:** In undifferentiated NSCs, FUBP1 induces the expression of c-myc. The c-myc interacts with JAK-STAT and is downregulated by SMAD/TGF beta pathway in gliogenesis. The c-myc is also involved in neurogenesis but plays the oncogenic role in GBM development. Cancer stem cells are heavily associated with the Wnt signaling. The dysregulated Wnt signaling causes the activation of cyclinD1 and c-myc, causing G1 to S phase transition. It also contributes to epithelial to mesenchymal transitions [190, 191].

#### Glioblastoma subtypes and vast heterogeniety

The Cancer Genome Atlas identified 840 genes involved in GBM, leading to the ultimate classification of glioblastoma into three subtypes [192–194].

1. **Pro-neural subtype:** This subtype is present mostly in younger patients. They tend to survive longer. It has the highest subtype shifting and most favorable survival [195]. IDH1 is one of the key genes involved in this subtype. It is also considered the reason for favorable prognosis. IDH1 mutant form is present with 1p/19q co-deletion [196]. Other important mutations include p53 dysfunction, PDGFR amplifications, upregulated Nkx 2-2, and Olig2. Most low grade glioblastomas and secondary glioblastomas belong to pro-neural subtype. Some most common GBM abnormalities such as chromosome 7 amplification and chromosome 10 deletions have low occurrence in this GBM subtype [197]. The EGFR, PTEN, and Notch are normal in this subtype [198].
2. **Classical subtype:** It is the most common subtype and key genetic alterations include EGFR amplifications, homozygous deletion of CDKN2A, and chromosome 7 amplification [199]. Abnormalities in IDH1, TP53, PDGFR, and NF1 are mostly absent in this subtype [200].
3. **Mesenchymal subtype:** It is the most stable subtype. Signature mutations include NF1, NF-κB, and upregulated gene expression of S100A1, CHI3L1, MET, VEGFR2, CD31, fibronectin, and COX2. There is increased inflammation and necrosis in this subtype [201].
4. There are fewer alterations of EGFR in this subtype. Pro-neural markers are dysregulated in this subtype. The alterations in TGF-beta and STAT3 play key roles in the transition from pro-neural to mesenchymal subtype. The proneural-mesenchymal transition upon tumor recurrence has been suggested as a mechanism of tumor resistance to multimodal therapy [196, 202].

The relationship between neurogenesis/gliogenesis and the key genes/signaling pathways involved in the subtypes has been investigated in the earlier sections.

### Significance of heterogeniety in glioblastoma

Glioblastomas are one of the most aggressive tumors with multiple subtypes and are widely known to have vast heterogeneity in nature. This heterogeneity is also evident among the pro-neural, classical, and mesenchymal subtypes of GBM. The genetic landscape of GBM is so diverse that there are lots of intra-tumoral heterogeneity as well. The landscape of GBM has vast heterogeneity that key epigenetic alterations such as those emerging because of IDH1/2 mutations are different in 3 subtypes and among different samples of GBM. Multiple studies have pointed out that landscape of GBM mutations vary to such an extent that RTK/RAS/PI3K alterations have been detected in 83% of samples, the TP53 alterations in 87%, and the RB1 alterations in 78% of the samples. Still there are lots of variability in even in core mutations among different samples [203–205].

There may be multiple factors that are responsible for this vast heterogeneity in GBM. Based on the results of this study, it is hypothesized here that this genetic variability may have origin in the differences between the genetic landscape of gliogenesis and neurogenesis. NSCs are the progenitors of both neurons and glial cells. The initial mutations that occur in three subtypes of GBM may drive the direction of subsequent mutations in tumor development. The internal genetic and epigenetic homeostasis that is established at the time of establishment of cell fate, when this homeostasis is dysregulated then the risk of glioblastoma oncogenesis increases. Aging plays a very significant role in GBM development as majority of the GBM cases are in old age. But the pro-neural subtype of GBM affects mostly younger patients and here different set of mutational landscape impacts GBM development. The timing and sequence of initial driver mutations also play very important role in altering the internal cell circuitry and direction of further mutational changes involved in GBM progression. An in-depth further discussion of GBM core pathways, heterogeneity, and subtypes is beyond the scope of this study.

## Discussion

### Cell type specification as the factor determining the risk of future oncogenesis

Neurons and glial cells both originate from neural stem cells (NSCs), despite this both are predisposed to different diseases. The number of tumors that originate in glial cells is so much higher comparatively. GBM is a grade-4 astrocytoma, while there are very few tumors that originate from neuronal cells such as medulloblastoma. The medulloblastoma mostly occurs in the young adults.

Aging plays a very crucial role in affecting glial cells and as well as neurons. The risk of neurodegenerative diseases including Alzheimer’s disease increases several fold in elderly, resulting in the massive degeneration of neurons. The risk of glioblastoma also increases several folds in elderly. Glial cells including astrocytes are considered to be major contributors to the GBM oncogenesis. By investigating genes and signaling pathways that are gliogenic and neurogenic, this study finds that the glial cells including astrocytes possess increased proliferative potential in comparison to neurons, and it predisposes them towards the increased risk of oncogenesis.

The cell circuitry in glial cells including astrocytes works in an integrated and deeply interconnected manner. For example, the EGFR is considered to be one of the key factors in GBM oncogenesis. In glial cells, the EGFR is upregulated along with ERK/AKT pathways in response to aging. There are many sets of upstream/downstream genes and signaling pathways which can be dysregulated by alterations in other related genes. Many such key genes are interconnected with JAK-STAT pathways which also exert control over other regulators of proliferation such as PI3K/AKT/mTOR pathway. Further cascade of pathological changes may lead to dysregulation of SOX2. This may contribute to enhance the stemness and invasiveness of GBM cells. This accumulation of dysregulations unfolds a cascade of changes that unleashes havoc in cell circuitry, ultimately leading to switching the cells towards oncogenesis. There are many gliogenic genes (JAK-STAT, hes, and others) and pluripotency-related genes such as FGF3, notch, wnt, and others, which in developmental mechanisms are very delicately regulated but they also possess the potential to contribute to the GBM oncogenesis upon being dysregulated.

While despite being the descendent of the same neural stem cells (NSCs), neurons and glial cells are very different cell types. Neurons are permanent cells with very limited and controlled expression of proliferation-related genes. It is because of the specific neurogenic genes that exert immense control over proliferative genes and signaling pathways to the extent that when their gene expression is induced, it also halts the proliferation in GBM cells. To a great extent, they also likely prevent neurons from the future risk of oncogenesis but predispose them more towards neurodegeneration in later life. The inverse relationship between Alzheimer’s disease and cancer biology has already previously been investigated by the author [4].

### Predisposition of glial cells towards the glioblastoma development

Similarly, like NF-κB signaling, the NRSF/REST gene expression is also upregulated with aging. This results in predisposing the cells towards more proliferative signaling. This genetic switching of the cells by the genes including NRSF/REST and others tend to predispose cells towards the increased risk of oncogenesis. The NRSF/REST expression has limitations in neuronal cells because they can interfere with neuron specific genes, but such limitations are not present in glial cells such as astrocytes. Hence, they become predisposed to increased risk of oncogenesis due to the nature of cell circuitry they possess. PAX6 expression in the maintenance of neuronal cells and its apoptotic effect in GBM tumor cells are also very important in signifying the impact of cell type specific genetic programming in disease predisposition. This also signifies how cell type-specific genes based on the nature of cell circuitry and cell type such as permanent or stable or labile determine the risk of future disease predispositions. For example, the risk of GBM in glial cells or the risk of Alzheimer’s disease-based neurodegeneration in neurons.

### Key neurogenic genes with ability to control oncogenesis in glioblastoma cells

***PAX6, Neurogenins including Ngn1, NeuroD1, NeuroD4, NKX6-1 Ebf, Myt1, ASCL1***

The role of Numb is controversial as it promotes neuronal differentiation and halts GBM oncogenesis, but its gene expression has also been detected in GBM mesenchymal cells.

### Key gliogenic genes with ability to control oncogenesis in glioblastoma cells

p300, BMP, PAX6 (anti-GBM override), HOPX (tumor suppressive + differentiation), NRSF/REST (astrcytogenic but capable of playing oncogenic role), LIF, and TGF beta: BMP is also involved in the neuronal development but it primarily has far greater gliogenic role. It is involved also in induction of neurogenin and ASCL1. Both are key regulators and have negative regulatory effects on GBM progression. The FGF signaling pathway also plays one of the key important and complex roles in gliogenesis. In GBM, FGF signaling pathway has been found to work with MAPK in GBM oncogenesis. But FGFR1 and FGFR2 also promote differentiation in glial cells.

There are around 350 gliogenic genes and 100 transcription factors that are involved in astrocytogenesis, but the GBM landscape is vast as it includes also the stemness related genes.

### Gliogenic vs. neurogenic programming of cells: determines the risk of disease predisposition in later life

Neurons are permanent cells while glial cells possess high proliferative capabilities. Such cell fate specifications based on cell types and their proliferative potentials act to determine the future disease predispositions. Neurons due to limited proliferative potentials are predisposed to the increased risk of neurodegeneration with aging, while astrocytes are at more risk of tumor development such as astrocytomas.

The key neurogenic genes including PAX6, neurogenins including Ngn1, NeuroD1, NeuroD4, NKX6-1 Ebf, Myt1, ASCL1, and Numb govern the neurogenic cell fate of neurons. They also decide the cascade of downstream genes which make neurons permanent cells by regulating cell cycle.

There is already age-related increase in gliogenesis, further increasing the risk of developing GBM. With aging, more and more NSCs begin to switch towards becoming astrocytes mediated via. STAT3. There is age-related decline in NSC pool. The result is more astrocytogenesis and less neurogenesis [206]. In gliogenic programming, around 350 genes and 100 TFs play role. Unlike neurons, glial cells are not permanent cell type. They have comparatively high capacity to proliferate and in cases of neuro-degeneration or stroke, gliosis is of common occurrence.

Cell type-specific programming of glial cells determines the gene expression of glial cells, and the euchromatin areas are predisposed to damage that may result in amplifications or deletions of genes. Overtime, this damage accumulates to the extent that cell cycle converges towards oncogenic G1 to S cell transitions in GBM, ultimately leading to stemness in GBM. The cell type specifications are not based on one gene but on combinations of genes and signaling pathways, hence called combinatorial code. Targeting any one gene such as EGFR fails to control the GBM oncogenesis and progression. This is because the dysregulations in one pathway or gene causes a cascade of downstream effects; hence, the rest of oncogenic circuitry remains intact.

GBM is considered a grade-4 astrocytoma. The genetic dysregulations of immense magnitude such as those contributed by REST, wnt, and shh are able to override the gene expression of other key genes whose purpose is to keep the cell in specific-differentiated state and to prevent uncontrolled proliferation. These dysregulations accumulate to contribute towards the progression of GBM. It results in the decline of cell type-specific-differentiated state and contributes to de-differentiation, increasing the grade and aggressiveness of tumor.

### GBM oncogenesis: a consequence of deviation from gliogenic-differentiated fate

Based on the findings of this study, this study postulate a possible sequence of key changes that unfolds and ultimately leads to GBM development. The risk of GBM increases several folds with aging. The origin of GBM is not based on the aberration of any one gene or signaling pathways. It originates as a consequence of and as accumulative effect of a wide variety of changes that dysregulate the cell circuitry of glial cells such as astrocytes. One example of such dysregulations include FGF pathway which also acts as neurogenic to gliogenic switch but when dysregulated it contributes to GBM development and becomes oncogenic.

In GBM oncogenesis, the initial sequence of oncogenic changes may vary as GBM occurs in sporadic manner and as well as part of other syndromes including in NF1, with loss of P53 in Li-Fraumeni syndrome and others [155, 207–209].

When dysregulations in cell circuitry exert enough control over the genes that keep cells differentiated in specific cell types (key gliogenic genes here), this leads to disruptions in the cell type-specific programming or combinatorial code that governs and maintains cell types in their respective differentiated states and maintains homeostasis.

### Possible landscape of GBM development

Here, we focus on the possible ways through which one oncogenic mentation in GBM leads to another and how interconnectedness of GBM circuitry may contribute to GBM progression. The GBM landscape varies depending on subtypes and intra-tumor heterogeneity. Here, we are postulating the GBM development landscape on the basis that how the emergence of oncogenic changes is capable of inducing further oncogenesis.

1. With aging, there is increase in levels of inflammation. The NF-κB gene expression becomes upregulated. This leads to the upregulation and amplification of EGFR.
2. EGFR amplifications contribute to the JAK-STAT dysregulations.
3. JAK-STAT dysregulations trigger a cascade of changes that further deviates the cells away from their differentiated state. This also leads to STAT3 dysregulations.
4. JAK-STAT pathway downstream targets include Bcl-xL, Bcl2l1, Bcl-2, cyclin D1, and c-Myc. The leads to the dysregulated STAT3 signaling.
5. BMPs which have major gliogenic role and also negatively regulate GBM. The EGFR amplifications also overcome the negative regulatory effect of BMPs.
6. The NF-κB also disrupts the notch signaling and alters the gene expression of cyclinD1.
7. Dysregulated Notch also causes simultaneous decrease in PTEN expression. This results in upregulation of Akt and VEGFR gene expression.
8. As PTEN controls SHH and PI3K, the PTEN dysfunction also makes both SHH and PI3K dysregulated. This causes the the dysregulation in PI3k/AKT/mTOR pathway.
9. SHH also has major interactions with key signaling pathways including TGF-beta, wnt/beta-catenin, notch, and also interacts with K-RAS, PKA, and others.
10. EGF motifs and Notch signaling are interlinked. The EGFR amplifications also disrupt the notch signaling. Notch signaling is also the regulator of EGFR, and this amplification is also modulated by dysregulations in TP53.
11. Notch also interacts with Hey1, PI3K/AKT/mTOR, and ERK/MAPK pathway. This contributes to the proliferative and survival signaling in GBM development.
12. NOTCH works with FGF to keep NSCs in proliferative stage. Both are dysregulated in GBM.
13. EGFR interacts with RAS-RAF-MEK-ERK and the PI3K-AKT-mTOR cascades and also interacts with CDKs.
14. EGFR amplifications also contribute to Pyruvate kinase M2 (PKM2) dysregulations, resulting in the upregulation of this rate limiting enzyme of glycolysis in GBM.
15. Dysregulations in EGFR and PTEN, also lead to the upregulation of Sox9 which contributes to further oncogenesis.
16. Dysregulated EGFR, FGF, PDGF, and c-MET also activate STAT 3 signaling. Dysregulated STAT3 contributes to upregulation of leukemia inhibitory factor receptor beta (LIF) which plays oncogenic role.
17. JAK-STAT pathway also has major interactions with TGF-beta which in normal glial cells prevent the cells from G1 to S transition. Dysregulations in JAK-STAT also impact TGF-beta.
18. Physiologically, TGF-beta regulates SOX2 and SOX4. Dysregulated TGF-beta may have far reaching consequences as SOX2 and SOX4 have been found to play role in GBM stemness.
19. SOX2 induces ASCL1 and TLX TF, and their dysregulation inhibits TGF Beta.
20. Sox2 is also interlinked with JAK-STAT signaling pathway, and both become dysregulated in GBM.
21. P300 is a very strong regulator of gliogenesis. It is also repressor of nestin which is also involved with stemness and interacts with Sox2. The c-myc gene overrides p300 and then GFAP. The leads to the upregulation of nestin.
22. TGF-beta regulates the cell cycle and cause the cytostasis by downregulating gene expression of c-Myc. This prevents G1 to S transitions. TGF-beta together with SMADs plays an important gliogenic role in normal development, but their dysregulated versions are oncogenic.
23. Dysregulated TGF beta also dysregulates PDGFR signaling.
24. Loss of tumor suppressors including p53 and PTEN, this loss also contributes to the GSK3 dysregulations.
25. GSK3 has interactions with many genes and pathways that are involved in GBM oncogenesis such as PI3k/AKT/mTOR pathway, wnt, notch, shh, ras, raf, mek, erk, APC, axin, sox2, and beta catenin.
26. In normal cells, the GSK3-beta acts as a negative regulator of EMT and many proto-oncogenes but dysregulated GSK3-beta is oncogenic.
27. REST gene: The direction of cell circuitry guided by it tends to have high proliferative potential. With aging, the gene expression of REST increases, further predisposing glial cells to oncogenesis.
28. REST also interacts with SHH, Wnt, and PI3K signaling pathways. All of them have well-established role in GBM oncogenesis.
29. REST has strong interactions with the wnt signaling pathways. It also downregulates the genes involved in apoptosis.
30. The dysregulated Wnt signaling causes the activation of cyclinD1 and c-myc, causing G1 to S phase transitions. Dysregulated WNT also contributes to epithelial mesenchymal transitions (EMT).

It is important to note that the landscape of all genes involved in GBM development is very vast. These abovementioned points focus on the key genes/signaling pathways based on the findings of this study to postulate the possible sequence of events through which GBM unfolds and develops. As the GBM landscape is vast, the sequence of mutations involved in GBM oncogenesis may vary but they always converge to cause the pathologic G1 to S transitions, ultimately leading to GBM development and progression.

### The targets for gene editing and epigenome editing in the development of future GBM therapies

The findings of this study also provide the targets for gene-editing tools such as CRISPR gene editing or epigenome editing to correct or regulate the genes/signaling pathways which become dysregulated in GBM development. This study may serve as a map for genetic and epigenetic targets for the development of new therapeutic approaches as it investigates the gliogenic and neurogenic genes/signaling pathways:

1. Having the ability to control oncogenesis in glioblastoma cells
2. Having gliogenic/neurogenic roles but in the GBM development contribute to the process of oncogenesis.
3. Having the ability to contribute to the gliogenesis or neurogenesis but also contribute to the stemness in GBM.

New potential therapeutic approaches may be devised with the goal to revert, halt, or control glioblastoma onset, development and progression by targeting-related gliogenic or neurogenic genes/signaling pathways as identified in this study.

## Conclusion

Glioblastoma originates when the gene expression of key gliogenic genes and signaling pathways becomes dysregulated. This study identifies key gliogenic genes/signaling pathways having the ability to control oncogenesis in glioblastoma cells including p300, BMP, PAX6 (anti-GBM override), HOPX (tumor suppressive + differentiation), NRSF/REST (astrcytogenic but capable of playing oncogenic role), LIF, and TGF beta.

It also identifies related key neurogenic genes/signaling pathways having the ability to control oncogenesis in glioblastoma cells including PAX6, neurogenins including Ngn1, NeuroD1, NeuroD4, Numb, NKX6-1 Ebf, Myt1, and ASCL1.

This study also postulates how aging contributes to the onset and origin of glioblastoma by increasing the gene expression of NF-κB, REST/NRSF, ERK, AKT, EGFR, and others. This is further detailed in the ‘Discussion’ section. It also evaluates how dysregulation of the key genes sets in motion a cascade of downstream changes that lead to the GBM oncogenesis. The origin of GBM is dependent on the multiple genes and pathways that accumulatively converge towards the disease development. There are multiple layers of steps in glioblastoma oncogenesis including the failure of cell fate specific genes (such as p300, BMP, HOPX, NRSF/REST, and others) to keep the cells differentiated in their specific cell type. The dysregulations in genes and signaling pathways (such as wnt, notch, shh) that are common to multiple cancers, also play significant role in GBM. The genetic regulators that are involved in pluripotency also become upregulated (such as sox2, oct4, c-myc), finally contributing to the development of cancer stem cells. They also have interactions with normal cell circuitry such as the interaction of Sox2 with JAK-STAT pathway. There is interconnected delicate balance of expression in the cell type-specific and survival-related genes. Such delicate balance is required for the maintenance of cell type and cell survival. When it becomes dysregulated beyond a specific threshold, it contributes to the development of glioblastoma. In GBM development, when the genetic dysregulations in key genes/signaling pathways that govern the cell fate and survival accumulate beyond a specific threshold, such dysregulations lead to the switching of cells towards the glioblastoma oncogenesis. Such mutations are capable of overriding the physiologic direction of cell cycle/circuitry by altering the gene expression of other gliogenic genes, proto-oncogenes, and tumor suppressors.

### Study design

The etiology and origins of glioblastoma (GBM) are not entirely known. This systematic study investigates the gliogenic and neurogenic genes/signaling pathways to trace the origins of glioblastoma. This research study finds evidence from the already published research literature to find the changes that lead to the onset and development of glioblastoma. This also will help to better understand the factors that predispose the glial cells more towards the risk of oncogenesis as compared to neuronal cells. The limitations are also explained in the ‘Methodology’, in the beginning of ‘Results’ section, and in other respective sections and headings.

### Limitations of the study

This study has cited studies based on the gliogenic and neurogenic genes/signaling pathway and has investigated them irrespective of GBM subtypes. The citation of studies was not based on any specific cell lines or specific tumor samples but was focused on evaluating the role of gliogenic and neurogenic genes in relation to their contributions in GBM oncogenesis. Hence, another limitation of this study is that it does not differentiate among the findings emerging from in vitro, in vivo, and in silico studies. We address this by including only those studies in this research work whose results were also evident by other independent multiple studies, as there are sometimes problems regarding reproducibility of the results from in vitro studies [210]. Due to the vast heterogeneity in GBM landscape and even in the same GBM sample, there is always possibility that one or more mechanisms present in one sample may be entirely absent in different tumor samples or subtypes [201].

In order to avoid overlooking unknown genes, this study takes a different approach and focuses on investigating the GBM development through the lens of gliogenic and neurogenic genes/signaling pathways. As the genetic landscape of gliogenesis and neurogenesis is very vast, hence it is not possible to focus on every gene in one study. There is always risk that other currently unidentified genes/signaling pathways may also be playing significant role in GBM oncogenesis. This study also tries to discuss the possible interconnectedness of genes/signaling pathways in GBM onset and progression in relation to the presence of specific mutations. The presence or absence of any mutation may alter the landscape of subsequent mutations. Mutational landscape also varies in different GBM subtypes and tumor samples.

This study divides the investigated gliogenic and neurogenic genes/signaling pathways into three categories as they were focused to investigate their role in GBM development.

1. Those having the ability to control oncogenesis in GBM cells.
2. Those having gliogenic/neurogenic roles but in GBM development contribute to oncogenesis.
3. Key genes that contribute to gliogenesis or neurogenesis but also contribute to the stemness in GBM.

## Data Availability

The datasets supporting this article are included within the article in the ‘References’ section.

## Abbreviations

GBM: Glioblastoma multiforme
NSCs: Neural stem cells
IL: Interleukin
CNTF: *Ciliary neurotrophic factor*
*LIF*: Leukemia inhibitor factor
CT-1: Cardiotriphin-1
FGF: Fibroblast growth factor
JAK: Janus kinase
STAT: Signal transducer and activator of transcription
SOX: SRY (sex-determining region Y)-box
BMP: Bone morphogenetic proteins
NIFA: Notch effector protein
EGFR: Epidermal growth factor receptor
GFAP: Glial fibrillary acidic protein
TGF beta: Transforming growth factor beta
CBP: CREB-binding protein
Hey1: Hairy/enhancer-of-split related with YRPW motif protein 1
HES1: Hairy and enhancer of split-1
DTX1: Protein deltex-1
SHH: Sonic hedgehog
PTEN: Phosphatase and tensin homolog
PAX: Paired box protein
Mek1: Mitogen-activated protein kinase
PKC: Protein kinase C
PI3K: Phosphoinositide-3 kinase
NPCs: Neural progenitor cells
CDKs: Cyclin-dependent kinases
PKM2: Pyruvate kinase M2
P53: Tumor protein P53
VEGF: Vascular endothelial growth factor
TF: Transcription Factor
NANOG: Homeobox protein NANOG
Wnt: Signaling pathway
Notch: Signaling pathway
GSK3: Glycogen synthase kinase 3
PDGFR: Platelet-derived growth factor receptor
KLF6: Krueppel-like factor 6
NF-κB: Nuclear factor kappa-light-chain-enhancer of activated B cells
EMT: Epithelial mesenchymal transition
Nrg1/Ngn1: Neuregulin-1
NRSF/REST: Neuronal restrictive silencing factor
MAPK: Mitogen-activated protein kinase
HOPX: Homeodomain-only protein
ASCL2: Achaete-scute complex homolog 2
NKX2-1: NK2 homeobox 1
mTOR: Mechanistic target of rapamycin
EFNB1: Ephrins
NTN: Netrins
NeuroD: Neurogenic differentiation factor
DBX2: Developing brain homeobox protein 2
Nkx-6.1: Homeobox protein Nkx-6.1
Myt1: Myelin transcription factor 1
Numb: Protein numb homolog
Oct2: Octamer transcription factor 2
Myc: MYC proto-oncogene
